# Attenuation of p53 mutant as an approach for treatment Her2-positive cancer

**DOI:** 10.1038/s41420-020-00337-4

**Published:** 2020-10-10

**Authors:** Olga Fedorova, Alexandra Daks, Oleg Shuvalov, Alena Kizenko, Alexey Petukhov, Yulia Gnennaya, Nikolai Barlev

**Affiliations:** 1 Institute of cytology RAS, St-Petersburg, Russia; 2 Almazov Federal North-West Medical Research Centre, St-Petersburg, Russia; 3 MIPT, Doloprudnuy, Moscow region, Russia; 4 Orekhovich Institute of Biomedical Chemistry, Moscow, Russia; 5 Chumakov FSC R&D IBP RAS, Moscow, 108819 Russia

**Keywords:** Breast cancer, Targeted therapies, Cancer therapeutic resistance

## Abstract

Breast cancer is one of the world’s leading causes of oncological disease-related death. It is characterized by a high degree of heterogeneity on the clinical, morphological, and molecular levels. Based on molecular profiling breast carcinomas are divided into several subtypes depending on the expression of a number of cell surface receptors, e.g., ER, PR, and HER2. The Her2-positive subtype occurs in ~10–15% of all cases of breast cancer, and is characterized by a worse prognosis of patient survival. This is due to a high and early relapse rate, as well as an increased level of metastases. Several FDA-approved drugs for the treatment of Her2-positive tumors have been developed, although eventually cancer cells develop drug resistance. These drugs target either the homo- or heterodimerization of Her2 receptors or the receptors’ RTK activity, both of them being critical for the proliferation of cancer cells. Notably, Her2-positive cancers also frequently harbor mutations in the TP53 tumor suppressor gene, which exacerbates the unfavorable prognosis. In this review, we describe the molecular mechanisms of RTK-specific drugs and discuss new perspectives of combinatorial treatment of Her2-positive cancers through inhibition of the mutant form of p53.

## Facts

- Her2-positive tumors are characterized by a high level of Ki67 expression, a worse prognosis of patient survival due to a high and early relapse rate as well as an increased level of metastatic potential.
- Only several drugs were officially approved by the U.S. Food and Drug Administration (FDA) for the treatment of Her2-positive breast cancer, known as trastuzumab, pertuzumab, trastuzumab emtansine (TDM-1), trastuzumab deruxtecan, lapatinib, neratinib, and pyrotinib.
- Mutant p53 protein induces overexpression of HER2.

## Open questions

- Can we use inhibitors of mutp53 for future treatment Her2-positive breast cancer?
- Is it important to detect the status of p53 in Her2-positive breast cancer?
- What approaches should be used to suppress mutp53?

## Breast cancer classification

Breast cancer, despite its heterogeneity, can be categorized into five subtypes: luminal type A (estrogen receptor (ER) and (or) PR positive, HER2 negative), luminal type B (ER and (or) progesterone receptor (PR) positive, human epidermal growth factor receptor 2 (HER2) positive), HER2 overexpressing (ER and PR negative, HER2 positive), triple negative or basal-like (ER, PR, and HER2 negative)^1^, and claudine-low breast cancer^2,3^ (Table 1). Based on immunohistochemical studies, pathologists subdivide the disease according to the expression status of receptors and growth factors, with particular attention being payed to the ER, PR, and Her2^2,4^. Her2-positive subtype occurs in ~10–15% of all cases of breast cancer. Her2-positive tumors are usually characterized by a high level of Ki67 expression, a worse prognosis of patient survival due to a high and early relapse rate as well as increased level of metastatic potential^2,5,6^. Out of those, 30–40% of breast cancer patients with overexpressed Her2 also have high levels of ER, while the rest exhibit diminished expressions of ER and PR hormone receptors, making them extremely resistant to targeting with antihormone therapy.

**Table 1:** Major molecular subtypes of breast cancer.

Subtype	ER	PR	Her2
Luminal type A	+	±	−
Luminal type B	+	±	±
Her2 overexpressing	−	−	+
Triple negative/basal-like	−	−	−

## Receptor tyrosine-protein kinase Her2 (erB-2)

Receptor tyrosine-protein kinase Her2 (ErbB2) is a member of the ERBB family of receptor tyrosine kinases (RTKs). The ErbB receptor family includes four members: ErbB1 (EGFR), ErbB2 (Her2), ErbB3, and ErbB4. ErbB receptors are localized on the cell membrane surface as inactive monomers. However, in the presence of ligands RTKs undergo homo- or heterodimerization, which changes their conformation and promotes autophosphorylation of their intracellular domains^7^. Ligand-induced conformational changes induce autophosphorylation on the tyrosine residue of the C-terminal domain, which serves as a platform for protein–protein interactions mediated by Src-domains resulting in the transfer of signaling through the PI3K/Akt pathways^8^. For example, Her2, besides homodimerization, can also form heterodimers not only with other members of the ErbB family, including EGFR^4,9,10^ but also with other RTKs, including insulin growth factor receptor (IGF1R) and c-Met. The latter event confers resistance to the receptor-specific inhibitory antibodies^11^. It is important to note that the extracellular domain of Her2 is always maintained in an open conformation ready to bind the activating ligands^10,12,13^. Notably, the specific ligand that can directly activate Her2 has not been identified so far^10^. Apparently, this promiscuity of Her2 in binding different partners and accepting various ligands allows tumor cells to actively proliferate.

## Her2-specific therapies

Her2, being the most important biomarker for several types of cancer, regulates such important cellular processes as proliferation, angiogenesis, cell adhesion and mobility, and also takes part in organogenesis and development^14^. Overexpression of Her2 or gene amplification occurs in 20–30% of cases of breast cancer and correlates with a poor prognosis of survival in patients. Anti-Her2 therapy is currently approved for Her2 overexpression in breast, stomach, and gastroesophageal cancers. However, the overexpression of Her2 has also been shown for other tumors found in places such as the bladder, cervix, colon, endometrium, glioblastoma, head and neck, liver, lungs, ovary, pancreas, and salivary ducts^15^. Her2-directed therapies include specific monoclonal antibodies that prevent the dimerization of Her2, such as trastuzumab and pertuzumab^15–18^, and/or small molecule inhibitors of its RTK activity to block the signaling cues, including lapatinib, neratinib, and pyrotinib^19^.

## Monoclonal anti-Her2 antibodies

**Fig. 1:**
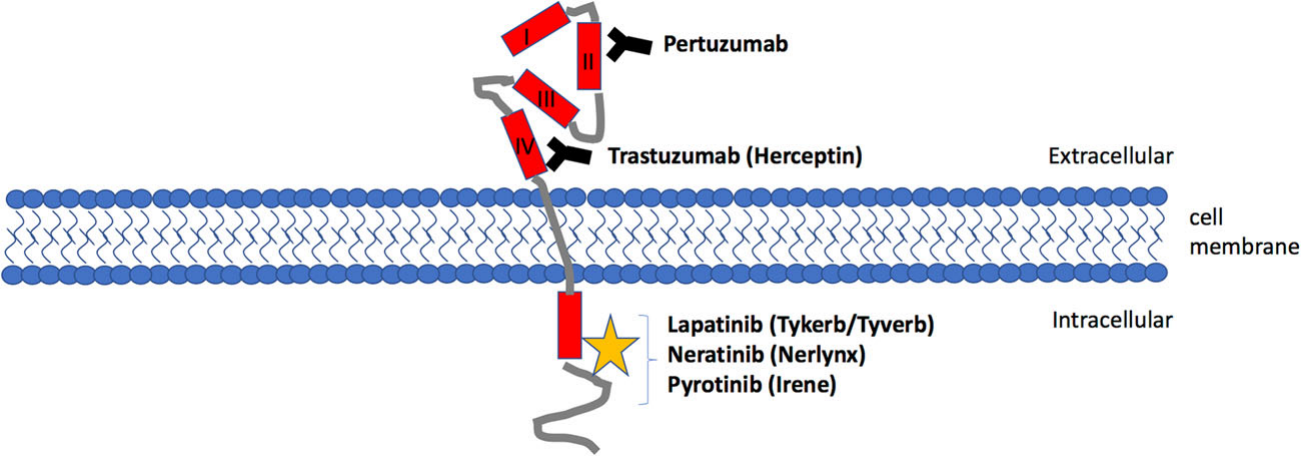
A scheme that illustrates major mechanisms of action by Her2-targeting agents. Antibody-based therapeutics pertuzamab and trastuzamab interact with the extracellular domain of Her2. Tyrosin kinasse inhibitors (lapatinib, neratinib, and pyrotinib) interact with the intracellular domain of Her2.

Trastuzumab (Herceptin), the first monoclonal anti-Her2 antibody, which was developed in 1990, inhibits Her2 signaling through several mechanisms: inhibition of dimerization; internalization and/or degradation of the receptor; inhibition of the PI3K-AKT signaling pathway; and antibody-dependent cell cytotoxicity (ADCC). Trastuzumab has been a standard treatment for both metastatic, and earlier stages of, Her2-positive breast cancer for more than 20 years^20–22^. Trastuzumab binds to the extracellular domain of Her2^23^. It is considered that the extracellular domain of the Her family of receptors consists of four subdomains (I–IV). Structural studies show that extracellular domains can be in two forms: tethered form through interaction II and IV subdomains and constitutively extended form through interactions between I and III subdomains^24^. It was shown that trastuzumab targets the IV subdomain of Her2^25^ (Fig. 1). Heterodimerization of Her2 with other RTKs blocks the access of trastuzumab to the IV subdomain domain thereby making the cells resistant to the therapy^26^.

Pertuzumab is a recombinant monoclonal antibody against Her2, the binding site of which is different from the site of trastuzumab. Pertuzumab binds to the dimerization domain (subdomain II of extracellular domain) of Her2, which leads to the inhibition of the heterodimerization of Her2 with Her3 (Fig. 1)^27–30^. Proliferation is suppressed by inactivating several downstream signaling pathways, including PI3K/AKT/mTOR and the mitogen-activated protein kinase pathway (RAS/RAF/MEK/ERK)^31^. In addition, to trastuzumab, pertuzumab triggers the ADCC reaction^32^. Although pertuzumab monotherapy has shown only moderate efficacy against Her2, a synergistic effect is observed when combined with trastuzumab^33^.

## Tyrosine kinase inhibitors

A wide range of tyrosine kinase inhibitors (TKI) have also shown a good level of efficacy in women with Her2-positive breast cancer. TKI interact with the intracellular catalytic kinase domain of Her2 (Fig. 1).

Lapatinib (Tykerb/Tyverb) acts as a double reversible inhibitor (TKI) of EGFR (Her1) and Her2, achieving a high overall inhibitory effect^34^. Lapatinib binds to the ATP-binding pocket of these receptors and hence prevents their self-phosphorylation, thereby blocking the activation of MAPK/Erk1/2 and PI3K/AKT downstream pathways^35,36^. Lapatinib has been shown to enhance Trastuzumab-dependent cellular cytotoxicity against breast tumor cells in in vitro studies^37^. Later studies have shown that lapatinib is metabolized by the P450 cytochrome system through 3A4 isozyme, resulting in the only metabolite activity against EGFR without targeting Her2^38^.

Neratinib (Nerlynx), an irreversible inhibitor of Her1, Her2, and Her4, which improves patient survival by 2-year when given to women with Her2-positive breast cancer after chemotherapy and adjuvant trastuzumab therapy^39^. Neratinib covalently binds to the ATP-binding site thus blocking the RTK kinase activity^40^.

Pyrotinib (Irene) is a novel irreversible anti-Her2 therapeutic drug that inhibits Her1, Her2, and Her4 by covalently binding the ATP-binding pocket of intracellular kinase regions^41^. A recent study of patients with HER2-positive relapsed or metastatic breast cancer showed that pyrotinib increased progression-free survival and objective response rates over lapatinib^42^.

## Antibody–drug conjugates

Trastuzumab emtansinem (adotrastuzumab) is an antibody–drug conjugate consisting of the monoclonal antibody trastuzumab bound to the cytotoxic agent mertansine (DM1), which is a tubulin inhibitor. This conjugate was designed to overcome trastuzumab resistance, which is developed in most Her2-positive patients and uses trastuzumab to deliver cytotoxic agent to Her2 overexpression cells. Adotrastuzumab is approved as the only treatment for Her2-positive metastatic breast cancer in patients who have already received trastuzumab and taxane individually or in combination. The approval is based on the results of a study, which compared adotrastuzumab with lapatinib and capecitabine. The study showed significantly longer survival without disease progression and overall survival with less toxicity than lapatinib plus capecitabine^43^.

Trastuzumab deruxtecan (Enhertu) is a novel antibody–drug conjugate recently approved by FDA for the treatment of metastatic Her2-positive breast cancer. This drug comprises of a monoclonal Her2-targeting antibody (trastuzumab) and a topoisomerase I inhibitor (deruxtecan) that showed very promising antitumor effects in preclinical xenograft models and clinical trials. This drug was designed to reach the cytotoxicity effect in tumor cells expressing Her2^44^.

However, none of these drugs can deliver a complete cure to patients, and eventually drug resistance develops in ~80% of HER2 + metastatic breast cancer patients^45^.

## Mechanisms of drug resistance

HER2-targeted therapies (trastuzumab, pertuzumab, TDM1, and lapatinib) are efficient at attenuating primary tumor progression and cancer relapse, but on the other hand, they apply a high selection pressure on the tumor to favor the clones with mutations in Her2 that become resistant to the therapy. For example, mutations in HER2 could perturb the antibody recognition or physical interaction between the drug and receptor. The T798M mutation in HER2 showed increased autophosphorylation activity and preference for heterodimerization with EGFR in human breast cancer cells. Interestingly, the treatment of Her2 T798M-expressing cells with an EGFR-specific antibody, cetuximab, reverted the trastuzumab resistance^46^. Similar to EGFR, which develops drug resistance to gefitinib by acquiring secondary mutations, Her2 also follows the same route. Specifically, in response to neratininb, mutant HER2 L869R was reported to acquire a secondary mutation at HER2T798I, reducing the binding between RTK and neratinib^47^.

Another mechanism of drug resistance to anti-RTK therapies in breast cancer is executed via the activation of other RTKs. For example, IGF1R is overexpressed in HER2+ breast cancer, and forms a heteromeric complex with HER2 and HER3 to activate the PI3K signaling pathway, conferring trastuzumab resistance to breast cancer patients^48^. Simultaneous treatment with anti-HER2 (trastuzumb) and anti-IGF1R mAbs (figitumumab) reportedly produced synergetic effects in breast cancer cells. Another RTK, c-Met, is frequently overexpressed in HER2+ breast cancer patients and similar to IGF1R contributes to trastuzumab resistance. Pharmacological inhibition of c-Met sensitizes the cancer cells to trastuzumab treatment^49^.

## The p53 family of tumor suppressors in breast cancer

Tp53 is one of the most important tumor suppressors in mammals. Being a transcriptional factor, p53 plays a key role in the maintenance of genome integrity by regulating such important processes as cell cycle progression, apoptosis, senescence, DNA repair, and cell metabolism in response to various forms of cell stress. Perhaps not surprisingly, the TP53 gene is the most commonly mutated gene in various tumors including breast cancer^50–53^. Importantly, about 30% of all breast cancer cases are characterized by mutations in the TP53 gene, while for HER2-positive subtypes, the proportion of TP53 mutations reaches 70%^54,55^.

The vast majority of p53 mutations occurs within the DNA-binding domain, which resides between amino acids 120 and 300. Only one-tenth of them leads to loss of p53 functions and the rest are missense mutations that result in the expression of a defective protein^56^. However, there are a few hotspot mutations, e.g., in the positions R248, R273, R175, and G245, that are enriched in breast cancer cells and are associated with an increased tumor growth rate, chemo- and radioresistance, and an unfavorable prognosis^57,58^. These mutations not only exhibit dominant-negative activity over wild-type p53 in case of heterozygosity but also confer new oncogenic properties to the protein, i.e., providing it with “gain of function” (GOF) features^50,59,60^.

Mutant p53 alters the gene expression program in various ways. One way that p53mut can affect transcription is through preventing p53 homologs, p63, and p73, from activating their target pro-apoptotic genes^61,62^. On the other hand, GOF p53 can act as a cofactor for other transcription factors such as NF-Y and ETS2, and promote cancer progression^50,63,64^.

Importantly, recent studies in mice have shown that GOF p53 (R172H, which is homologous to the human R175H mutant form) facilitates p53LOH after DNA damage^65^. This means that genotoxic therapy administered to breast cancer patients with such mutations in the TP53 gene will eventually result in the ablation of the wild-type copy of TP53 and worsen the survival prognosis. Furthermore, the p53 GOF forms were shown to be more stable on the protein level in cancer cells compared to their normal counterparts^66,67^. The main mechanism responsible for the degradation of both p53wt and p53mut is through the ubiquitin-dependent proteasome system^67,68^. MDM2 is the main p53-specific E3 ubiquitin ligase that targets the p53 protein for proteasomal degradation^69,70^. Being a direct transcription target of p53, Mdm2 expression is attenuated in the presence of mutant p53 and hence the latter is accumulated in the cell^71^. Furthermore, upon DNA damage, p53 undergoes multiple post-translational modifications, which compete with MDM2-mediated ubiquitination, thereby indirectly stabilizing the p53 protein^72–74^. On a related note, mutant p53 is constitutively phosphorylated on Ser15 by ATM in the human breast carcinoma cell lines MDA-MB231 and MDA-MB468^75–77^. Moreover, chaperones HSP70 and HSP90 and co-chaperones BAG contribute to the maintenance of mutant forms of p53 in cancer cells via their interactions with mutp53 and hence preventing its proteasomal degradation^78,79^. Importantly, in response to DNA damage proteasomes themselves undergo post-translational modifications that affect their activity and composition^80–82^. Taken together, post-translational modifications play a very important role in the regulation of p53. Future experiments should clarify how various covalent modifications affect the interactome of mutant forms of p53 thereby affecting their functions.

Since GOF mutant forms of p53 are more stable in cancer cells, it is not surprising that strong p53 signals obtained by immunohistochemistry are associated with more aggressive forms of cancer and worse survival prognosis for patients.

## Analyzing the association of p53 and Her2 in breast cancer

In breast cancer patients, the majority of Her2-positive cases harbor p53 mutations, which correlate with a poor prognosis. Also, women patients with p53 germline mutations (Li–Fraumeni syndrome) have been shown to have a significantly higher probability (83%) of developing breast cancer with Her2 overexpression^83^. Analysis of these patients showed that tumors with a higher Ki67 proliferation index were associated with greater Her2 overexpression, larger size, and greater lymph node damage compared to other types, and are considered more aggressive^84^.

A relationship between p53 mutations and the early onset of Her2-positive breast cancer has been demonstrated^85^. These data indicate an indirect association of p53 and Her2 expression. In the study of Ferrari et al. the authors divided 64 Her2-positive breast tumors into four subgroups (A, B, C, and D) with subgroups C and D overexpressing Her2. They found that primarily these C and D subgroups were enriched with p53 mutations^86^.

**Fig. 2:**
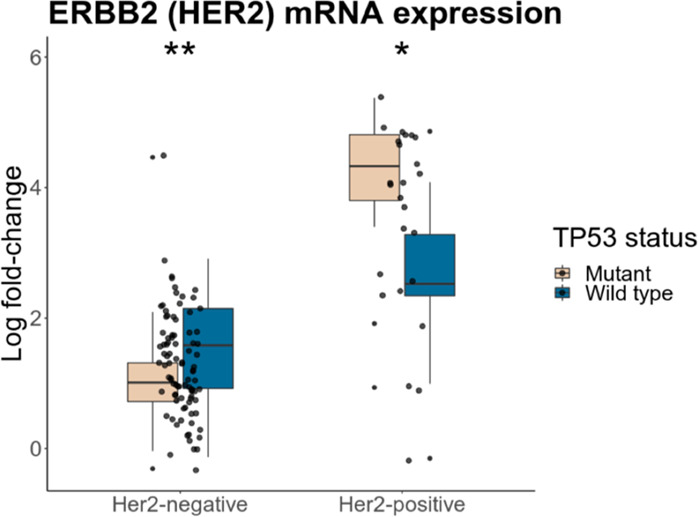
Boxplots demonstrating ERBB2 (Her2) gene expression levels depending on the status of p53 in Her2-negative and Her2-positive tumors. Her2-negative tumors with mutp53 (beige) were compared to Her2-negative tumors with wild-type p53 (blue). Her2-positive tumors with mutp53 (beige) were compared to Her2-positive tumors with wild-type p53 (blue).

To obtain more insights into the relation between Her2 expression and the p53 mutation status in breast cancer patients we performed an analysis of the breast cancer dataset (GSE22358) from the public GEO database. ERBB2 (Her2) gene expression levels from breast cancer patients were analyzed using a Wilcoxon–Mann–Whitney statistical test to evaluate Her2 gene expression differences in tumor tissues with a different p53 status (Fig. 2). In Her2-positive tumors, Her2 mRNA levels are significantly higher in samples with mutant p53 (*p* value = 0.046) comparing to samples with wild-type p53. On the other hand, in Her2-negative tumors, the Her2 mRNA level is significantly lower in samples with mutant p53 (*p* value = 0.0024) compared to samples with wild-type p53. Collectively, our data indicate at an association between the presence of mutant p53, but not wild type, and Her2 expression.

In the mouse model, mutp53 R172H was shown to interact with oncogenic Her2 signaling in the development of breast cancer. The authors found that mutp53 enhanced Her2/EGFR signaling, thereby facilitating the proliferation of breast cancer cells and increasing the population of breast cancer stem cells^87^. Mechanistically, mutp53 can increase the level of Her2 twofold: first, mutp53 can enhance the transcriptional activity of HSF1 (heat shock transcription factor 1), whose target (among several others) is chaperon Hsp90, which in turn, stabilizes Her2 and mutp53 itself on the protein level^88^. Importantly, double Her2/EGFR inhibitor, lapatinib, inhibits the Her2–HSF1–mutp53 interaction, which leads to the destabilization of mutp53 protein in cancer cells^89^.

The second mechanism of Her2 regulation by mutp53 was shown by Roman-Rosales et al.^90^ The authors showed that the transfection of mutant forms of the p53 protein (R248Q and R273C) in cell lines led to an increase of Her2 expression. Conversely, suppression of mutp53 reduced the expression of Her2 on the level of transcription^90^. Thus, the status of p53 affects the expression of Her2 in opposite ways, either promoting the proliferation or apoptosis of cancer cells^91^.

## Perspective therapeutic approaches to treat mutant p53- and Her2-positive cancers

The wealth of accumulated experimental data strongly suggests that combination therapeutic approaches are more efficient in targeting solid tumors compared to monotherapy. Following these logics, it would be reasonable to assume that different combinations of Her2-specific drugs combined with anti-mutp53 therapy would give the best result. Unfortunately, not many chemicals that would specifically target mutant p53 are available today. In this respect, high expectations are associated with a new experimental drug, APR-246, which reverts the mutant conformation of p53 to its wild-type form^92^. It would be interesting to see whether the combination of APR-246 and Her2 inhibitors produce a synergistic effect on killing the breast cancer cells.

Besides targeting mutp53 directly, there are several alternative approaches. For example, it has been shown that the mutant forms of the members of the p53 family increase the immunogenicity of cancer cells^93,94^. Bioinformatic analysis revealed that almost all analyzed immune signatures showed significantly higher enrichment levels in TP53-mutated breast cancer cells compared to wild-type TP53 cells^95^. Taken these results together, it seems as a viable approach to test immune therapy on Her2-positive/mutp53 either with specific CAR-Ts or using anti-PDL1/CTLA4-therapy.

One of the most common forms of mutp53 expressed in Her2-positive breast cancers is GOF p53 R248Q p53. Since this protein assumes mutant conformation, its stability is critically dependent on the presence of Hsp90 chaperon. Accordingly, Schulz-Heddergott et al. have shown that Hsp90 inhibition reduced the level of mutp53 R248Q and inhibited colon cancer progression. In this respect, it would be interesting to see whether the same approach, when combined with Her2 inhibitors, inhibits the growth of breast cancer cells^96^. Supporting this finding is the recent report demonstrating that Hsp90 inhibitor 17-AAG eliminates R248Q by stimulating macroautophagy under normal growth conditions^96^.

**Fig. 3:**
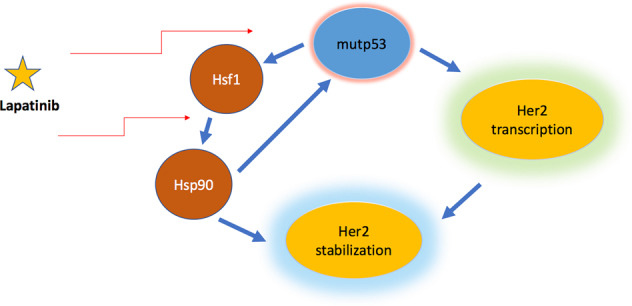
A scheme that depicts the potential mechanism of how mutp53 affects the expression of Her2. Mutp53 can influence transcription level of Her2, and as a result stabilize Her2. Mutp53 physically interacts with and enhances the transcriptional activity of HSF1, which is the main transcriptional regulator of Hsp90. Hsp90 stabilizes Her2 and mutp53 itself. Moreover, Her2 inhibitor lapatinib inhibits the ternary Her2–HSF1–mutp53 interaction, which leads to the destabilization of the mutp53 protein in cancer cells.

Work by the group of Del Sal^97^ and by others^98^ highlighted that statins, a class of MVA pathway inhibitors and a very common drug used in the clinic for treatment of cardiovascular diseases, elicit mutp53 destabilization and reducing cancer cell proliferation. Future experiments will assess the validity of our hypotheses (Fig. 3).
